# Layer-by-Layer Nanocoating of Antiviral Polysaccharides on Surfaces to Prevent Coronavirus Infections

**DOI:** 10.3390/molecules25153415

**Published:** 2020-07-28

**Authors:** Daniel P. Otto, Melgardt M. de Villiers

**Affiliations:** 1 Research Focus Area for Chemical Resource Beneficiation, Laboratory for Analytical Services, Faculty of Natural and Agricultural Sciences, North-West University, Potchefstroom 2531, South Africa; 2 Division of Pharmaceutical Sciences–Drug Delivery, School of Pharmacy, University of Wisconsin-Madison, 777 Highland Ave, Madison, WI 53705, USA; Melgardt.deVilliers@wisc.edu

**Keywords:** layer-by-layer nanocoating, antiviral polysaccharide, green chemistry, protective masks, work surfaces

## Abstract

In 2020, the world is being ravaged by the coronavirus, SARS-CoV-2, which causes a severe respiratory disease, Covid-19. Hundreds of thousands of people have succumbed to the disease. Efforts at curing the disease are aimed at finding a vaccine and/or developing antiviral drugs. Despite these efforts, the WHO warned that the virus might never be eradicated. Countries around the world have instated non-pharmaceutical interventions such as social distancing and wearing of masks in public to curb the spreading of the disease. Antiviral polysaccharides provide the ideal opportunity to combat the pathogen via pharmacotherapeutic applications. However, a layer-by-layer nanocoating approach is also envisioned to coat surfaces to which humans are exposed that could harbor pathogenic coronaviruses. By coating masks, clothing, and work surfaces in wet markets among others, these antiviral polysaccharides can ensure passive prevention of the spreading of the virus. It poses a so-called “eradicate-in-place” measure against the virus. Antiviral polysaccharides also provide a green chemistry pathway to virus eradication since these molecules are primarily of biological origin and can be modified by minimal synthetic approaches. They are biocompatible as well as biodegradable. This surface passivation approach could provide a powerful measure against the spreading of coronaviruses.

## 1. Introduction

In this paper, the role of a layer-by-layer nanocoating approach to provide a mechanism of prevention of the spreading of the corona- and other viruses is described. The emphasis is placed on passive prevention techniques that may contribute to curbing the spread of the pathogen, rather than active pharmaceutical measures. It must be emphasized that the continued efforts toward vaccination and pharmacotherapeutic measures are essential and should continue. It is suggested that our non-pharmaceutical prophylaxis measures will aid the pharmaceutical measures in a complementary fashion.

The perspective provided here is that several measures can be taken to prevent the spreading of the pathogen and attempt to combat the virus before it even enters the body. Despite our description of external measures against coronaviruses, it still relies significantly on the knowledge gained by renowned researchers who have established and studied vaccination and pharmacotherapeutic interventions against viruses in general.

## 2. The Cell Entry Mechanism of Encapsulated Viruses

Encapsulated viruses such as the SARS-CoV and SARS-CoV-2 viruses comprise of some general surface constituents. The surface envelope or capsule is presented as a lipid bilayer membrane that contains various envelope proteins (E), membrane proteins (M), and an outer layer that presents so-called spike (S) proteins [1]. M and S proteins are generally rich in sugar molecules that form a so-called glycan structure. *N*- or O-glycosylate moieties are commonly found in the viral S proteins and they can recognize some cell receptors to which the virion can bind [2,3]. These spike proteins facilitate virion entry into host cells. Encapsulated viruses such as the coronaviruses present approximately 200 of these spiky structures [4]. Spike proteins are comprised of glycoproteins, proteins that also contain polysaccharide or oligosaccharide moieties otherwise known as glycans [5,6,7].

The glycoproteins have a variety of functions that maintain the virion structure and properties such as water solubility, creation of diffusion barriers, and antiadhesive actions among others [6]. In addition to the intrinsic functions that glycoproteins afford to the maintenance of the virion structure, they also act as a structure that recognizes glycan-binding proteins presented on the membranes of potential host cells [1]. The viral glycans may be recognized by bacterial, fungal, and parasite-associated glycan-binding proteins. However, viruses are also recognized by host cells via the same mechanism. It is this form of intercellular recognition interactions that prove vital to effect the virus entry into host cells in which the virus could replicate [7].

A detailed description of the spike glycoproteins of SARS-CoV-2 reported that two binding subunits can be distinguished. These subunits become active when the two units are cleaved by host cell proteases on the host cell membrane. Subunit, S_1_ is responsible for binding to the host cell membrane and subunit, S_2_ is responsible for fusion of the virion and host cell membranes. The S_1_ unit is the factor that makes various coronaviruses specific toward a certain host [8]. Pulmonary angiotensin-converting-enzyme 2 (ACE-2) in humans exhibit the appropriate receptor, a specific sequence of amino acid residues [9], towards S_1_ and partly explains the effective spread of the coronaviruses via droplets in the atmosphere [10].

**Figure 1:**
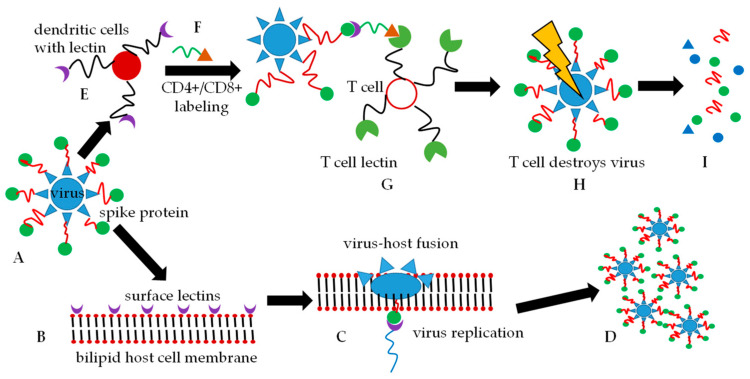
Two simplified routes of the fate of an encapsulated virus are shown. Either route (**A**–**D**) or route (**A**–**I**) can be followed. (**A**). The virus with spike proteins comprising of *N*-glycan moieties on the protein (red and green) is presented. (**B**). A potential host cell presents glycan-recognizing lectins on its bilipid membrane surface. (**C**). The virus glycan array binds to the host cell lectins and membrane fusion is initiated and after phagocytosis, virus replication follows. (**D**). Host cell destruction takes place with the subsequent release of new virus particles. (**E**). The virus is intercepted by dendritic cells before it can interact with the host cell membrane. The dendritic cells label the virus with cytokines CD4+/CD8+ (green and orange symbols), and (**G**). presents the cytokine-labeled virus to T-cells. (**H**). T-cells recognize the CD4+/CD8+ labels and phagocytose the virus that is destroyed in the T-cell lysosomes. (**I**). Only inactive, non-pathogenic viral degradation products remain.

As part of the human host immune responses, the glycans of the coronavirus spike protein subunits are recognized by dendritic cells [11] in the blood which binds to the glycan and subsequently expresses CD4+ and CD8+ glycopeptides. These glycopeptides label the spike protein and this labeled protein is then presented to T-cells [12]. T-cells subsequently recognize the labels, phagocytose these antigen-marked viruses, and degrade them. It has been found that the glycan-binding proteins, also known as lectins [5], can impart broad-spectrum binding properties against HIV-1, SARS-CoV, and human cytomegalovirus. The lectin which is capable of showing broad interaction via oligomannosyl antigens is known as lectin GNA (*Galanthus nivalis* agglutinin). The *N*-oligomannosyl cores are embedded in *N*-glycans which are commonly expressed on the surface of numerous viral pathogens [13]. Once the lectin binds to the glycan, the virus structure may undergo conformational changes that result in the fusion of the virus and host to facilitate virus entry. S-proteins are specifically responsible for host cell entry by coronaviruses [14]. Figure 1 depicts a simplified entry mechanism of the viruses into host cells.

## 3. Potential Targets for Drug Development against Coronaviruses

It has been shown at the time of writing that ACE-2 receptors in the lungs are specific targets for SARS-CoV-2 spike proteins [15]. It has been found that lung parenchyma must be in the differentiated state of the cell cycle and must express the ACE-2 receptor on the apical side, i.e., the site of the first contact with air, of the parenchyma [16,17,18,19]. It is no surprise therefore that the SARS, MERS, and SARS-CoV-2 viruses cause significant lung injury [20,21,22,23,24]. ACE-2 is also expressed to a significant extent in the gastrointestinal tract (GIT) [25] which accounts for the GIT symptoms that appeared in SARS-CoV patients and currently in SARS-CoV-2 infected patients [26].

Current pharmaceutical efforts against coronaviruses aim to interfere with the binding of the viral glycans in their spike proteins to potential host cell receptors. The drug umifenovir has been shown to prevent the fusion of viral S-proteins with the host cell ACE-2 receptors, therefore excluding cell entry of the virus. However, umifenovir was clinically inefficient to improve the Covid-19 disease outcome [27].

A contributing membrane-bound enzyme that facilitates spike protein entry via ACE-2 receptors is the TMPRSS2 serine protease. This protease primes the coronavirus spike protein for entry by cleaving protein domains that increase the interaction with host cell membranes that result in membrane fusion [28]. It was also suggested that TMPRSS2 cleaves ACE-2 sites that promote the affinity for the viral spike protein, hence enhances cell entry [29].

Another possible target for treatment is the inhibition of the synthesis of the required saccharides that are incorporated in the host cell glycoproteins. Several quinine derivatives showed that these saccharide moieties known as sialic acids could be inhibited by drugs such as hydroxychloroquine. The quinines, therefore, decrease the number of glycan targets that could be engaged by coronaviral spike proteins to facilitate cell entry [30,31,32]. However, quinine derivatives pose significant cardiotoxicity that precludes their application as a treatment for Covid-19 [33].

**Figure 2:**
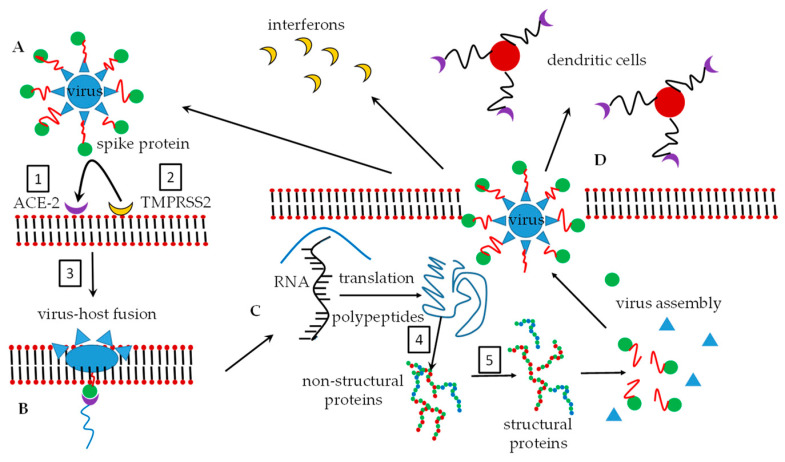
Potential targets for drug therapy. (**A**). The virus spike proteins interact with ACE-2 receptors and this provides the route of entry of the virus into the host cell. 1. Drugs such as arbidol interfere with the binding of spike proteins with ACE-2 receptor. 2. Priming of the spike protein to enhance its affinity for ACE-2 receptors is hampered by, for example, camostat. 3. Virus-host membrane fusion could be prevented by drugs such as chloroquine. (**B**). The successful fusion of the virus and host membrane is achieved. (**C**). The coronavirus sheds its RNA which can then be translated to polypeptides. The polypeptides as cleaved by the enzyme, 3-chymotrypsin protease to render non-structural proteins. This proteolysis step, **4**, can be inhibited by, for example, lopinavir. Drug target **5**, prohibits the further conversion of non-structural to structural proteins by RNA-dependent polymerase enzymes. Remdesivir is a prime example of an inhibitor of target **5**. (**D**). Virus assembly has been completed and the virus is expelled from the host cell. The virus can then be intercepted by dendritic cells or other immune factors such as interferons (not discussed) or restart the replication cycle. Figure adapted from [40].

Antiretrovirals such as nelfinavir have proven to be efficient inhibitors of post-entry replication of the virions of SARS-CoV [34,35]. It has been found that protease inhibitors used in SARS and MERS studies prevented the cleavage and activation of spike proteins following the translation of precursor polypeptides from the viral RNA [36,37]. RNA is a vital step towards the ultimate formation of the structural protein of new viruses [34]. Ritonavir and lopinavir [38] have shown some good activity against the SARS-CoV protein cleavage that ultimately prevents spike proteins from forming. Some investigated drugs have been reviewed as protease inhibitors against coronaviruses and the reader is referred to the publication by Adedeji et al. [39]. Figure 2 shows the currently identified targets for which drugs are investigated.

Vaccination efforts are attempting to create a substrate that can be recognized by dendritic cells. The dendritic cells will subsequently label the virus spike proteins with coronavirus specific CD4+/CD8+ peptides. These specifically loaded particles will then be presented to T-cells that interact with T-lymphocyte dependent antigens. This interaction results in the induction of B-lymphocytes that produces antibodies that can recognize the spike proteins of coronaviruses to afford phagocytosis and destructions of the virus. It is also expected that memory T-cells will form which could result in long-term immunity when these cells are replicated when a spike protein can be recognized by dendritic or other immune cells. The carbohydrate-based vaccine holds promise to present recognizable glycan arrays that are representative of the targeted pathogens [41].

Very recently, several targets have been identified for the development of SARS-CoV-2 vaccines that have been identified based on the similarity of the virus to a previously encountered coronavirus, SARS-CoV [42]. It has been proven that many of the human pathogenic coronaviruses share a significant genomic overlap of more than 99.9% and this provides the basis for developing vaccines from a previously encountered coronavirus infection before SARS-CoV-2 [15,43]. The Middle East respiratory syndrome-related coronavirus, MERS-CoV, and SARS-CoV form part of these β-coronaviruses which wreaked havoc in the last two decades [44].

Therefore, one of the most promising targets is the characterization of spike protein epitopes that can be recognized by immune cells such as B- and T-cells. Some form of replication of this spike protein epitope should, therefore, be developed and this will hopefully provide the antigens which can bind to, for example, B- or T-cell antibodies and alert the immune system to coronaviruses [44,45]. A recombinant vaccination approach could be demonstrated by expressing the receptor-binding domain [46] of SARS-CoV spike protein in an adenovirus. This recombinant adenovirus was administered nasally and T-cells showed a significant immune response towards the expressed spike protein-binding domain [47].

Recombinant DNA approaches have made significant strides towards the synthesis of peptide sequences that can directly bind to spike protein epitopes. Spike proteins of the MERS-CoV were neutralized in this way [48,49]. The spike protein antigen of the MERS-CoV was also reported and identified targets for spike protein deactivation [50].

It is emphasized that the discussion of chemotherapeutic and vaccination efforts is superficial and not covered in detail in this report. Of importance though is the fact that antigen-antibody interactions are a key interest in developing vaccines. This recognition mechanism is creating the prelude for the discussion of the layer-by-layer nanocoating process that will be elaborated in due course.

## 4. The Role of Polysaccharides in the Treatment of Coronavirus Infections

Mammalian cell membranes are decorated by glycoproteins that contain glycans or polysaccharides. As described above, polysaccharides and proteins can interact and if the interaction happened to be between matching polysaccharides and proteins [51], a viral infection, for example, will take place [52].

Antiviral polysaccharides can, therefore, be very efficient against viral, and other pathogenic, infections if appropriate polysaccharide moieties can be exploited for protein recognition. Through recognition of the pathogen mucopolysaccharides, or glycosaminoglycans (GAG), have significant potential to explore drug leads. GAG is expressed ubiquitously throughout the body on cell surfaces as well as in the extracellular space between cells. An interesting property of these polysaccharides is that they are very diverse in their structure and also not monodisperse, precisely defined molecules. Various enzymes are capable of modifying these polysaccharides at various bodily sites to perform a particular function [53].

GAG is distinguished into four categories namely heparin/heparin sulfate, chondroitin/dermatan sulfate, keratan sulfate, and hyaluronan. One can deduce that the GAG biopolymers are sulfated, except for hyaluronic acid [54], and therefore negatively charged.

GAG is a class of naturally occurring polysaccharides that are commonly described as mucopolysaccharides. Mucopolysaccharides are commonly found in the mucous and act as viscous lubricants [55]. Mucopolysaccharides were traditionally considered to be inert, however, of late have been increasingly more recognized for their immunomodulatory action due to the multipotent recognition of various lectins [55,56,57] or specific protein recognition that often forms the basis from polysaccharide drugs are derived for broad-spectrum antiviral activity [58].

GAG can also be tailored to elicit specific bioactivities. Reported specific examples include the modification of heparin-like structures against for example dengue and flaviviruses [59], herpes simplex 1 and 2 viruses [60]. GAG-derived drugs can act as a decoy target for viral recognition [61] and therefore prevent cell membrane interaction that will allow virus entry into the host cell [62]. They also facilitate cell adhesion, cell growth, and differentiation, as well as cell signaling and anticoagulation [55].

Since these molecules are polymers, numerous sites for binding interactions exist and pathogens might even be capable of incorporating host cell glycans [9]. Coronaviruses have been known to interact with heparin sulfate proteoglycans, one of the most commonly occurring glycoproteins in eukaryotic organisms, and facilitate virion adsorption to cell membranes [63]. The spike protein of SARS-CoV-2 is shown to interact with heparin which is derived from the GAG, heparan sulfate and this indicated a potential target for heparan and heparin-derived drugs against SARS-CoV-2 [64,65,66,67]. Heparan sulfate is a promising target since heparin glycans are widely expressed in lung cells. Heparan sulfate is an important co-receptor for ACE-2 since ACE-2 receptor expression is too low to directly result in infection. SARS-CoV [68,69] and CoV-NL63 [70] coronaviruses initially bind to heparin sulfate which concentrates the virion concentration close to the ACE-2 receptor for cellular entry.

Studies involving other SARS-CoV-2-unrelated viruses such as the Ebola virus [71], human metapneumovirus [72], and Ross River virus [73] have also illustrated that the heparan sulfate is a common glycan receptor for viral proteins. It is strongly suggested that an electrostatic interaction is to blame for the interaction between negatively charged sulfate groups of GAGs and positively charged arginine amino acid residues of the virion binding S_1_ subunit responsible for virion adsorption. CoV-NL63 [74], hepatitis E virus [75], capsid proteins of adeno-associated virus type 2 [76], and swine fever virus [77] are but a few viruses that indicated cationic electrostatic interaction with negatively charged heparan sulfate.

It was again found that significant attention is garnered by the development of polysaccharide-derived drugs. The focus now shifts on these examples, however, one has to recognize the protein-glycan interaction between pathogen and host cell membranes. It is also apparent from the literature that sulfated GAGs are the most potent receptors for a huge variety of virus proteins.

The occurrence of electrostatic interactions between the spike proteins of coronaviruses, among others, and the prominent GAG, heparan sulfate introduces the discussion on layer-by-layer (LbL) nanocoating.

## 5. What Can LbL Nanocoating Contribute to the Prevention of Infectious Disease?

### 5.1. The Process of Layer-by-Layer (LbL) Nanocoating

LbL self-assembly of polyelectrolytes took its origin in the 1990s [78]. Poly(styrene-4-sulfonate), PSS, was one of the first polyanions employed for LbL self-assembly and remains widely utilized today. As polycation, an ammonium-containing polymer, poly(*N*,*N*-dimethylallylamine), PDDA, was successfully employed to create a multilayer structure comprising of alternating polyanion and -cation layers [79].

A series of proteins were also successfully employed as polycations namely cytochrome c, lysozyme, histone f3, myoglobin, and hemoglobin. By adjustment of the pH of the medium, amylase, glucose oxidase, and catalase were employed as polyanions [80]. DNA was also employed successfully as a polyelectrolyte for LL self-assembly [81].

LbL coating has also been employed to modify inorganic surfaces. Although many applications for these surface modifications are possible, only some antimicrobial examples are mentioned. Stainless steel surfaces were primed with an acrylate-based surfactant via electrografting. Subsequently, PSS and PDDA layers were coated in an alternating fashion. Lastly, a layer of chitosan was coated as an antibacterial layer against *E*. *coli* and *S*. *aureus* [82]. Silicone-based intraocular lenses (IOL) are commonly employed to replace the natural eye lens when it is damaged. The IOL can, however, allow adhesion of many kinds of bacteria and lead to post-operative infections with catastrophic effects in some patients. LbL nanocoating of the lenses with hyaluronic and chitosan had significant anti-adhesion and bactericidal effects that reduced the risk of postoperative infections [83,84].

**Figure 3:**
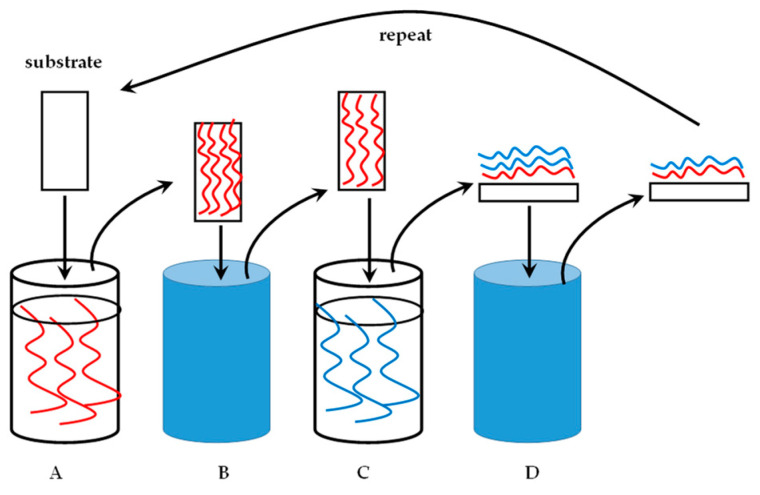
A substrate undergoing layer-by-layer (LbL) nanocoating. (**A**). Polyelectrolyte solution with a specified charge in a dipping container. The substrate is immersed in this solution for a predetermined time. (**B**). The coated substrate is placed in water to wash off the excess, unbound polyelectrolyte solution. (**C**). The washed, coated substrate is immersed in a polyelectrolyte solution of an opposite charge relative to the first solution. (**D**). A bilayer of the polycation and -anion is formed. The excess of the second polyelectrolyte (blue) is washed off to produce a substrate with a single bilayer of the polyelectrolytes as a nanocoating. The process is repeated for the desired amount of cycles.

The technique of LbL nanocoating is uncomplicated and requires relatively low concentrations of the polyelectrolytes to produce an efficient coat, often in the low nanometer range. A substrate for coating is required, a polycation and separate polyanion solution, and clean water as the washing liquid. Figure 3 illustrates the technique. Numerous polysaccharides, especially GAGs, are charged polyelectrolytes and the next section will elaborate on this.

### 5.2. Employment of Polysaccharides as LbL Materials

In this paper, the focus will fall only on common GAGs and other common polysaccharides such as chitosan. It was also noticed during our literature survey that the GAG, keratan sulfate has not been studied in LbL applications and can most probably be attributed to its production in the cornea, cartilage, and bone tissues which makes it fairly inaccessible. To date, the GAGs and other polysaccharides have not been employed widely in LbL nanocoating to specifically produce antiviral surfaces as is the case for antibacterial or antifungal coatings. Numerous publications have reported on the antibacterial surface application of polysaccharides via an LbL approach [85,86,87,88,89,90]. Table 1 lists some commonly utilized polysaccharides that have been employed in LbL nanocoatings and a non-exhaustive list of recent applications.

**Figure 4:**
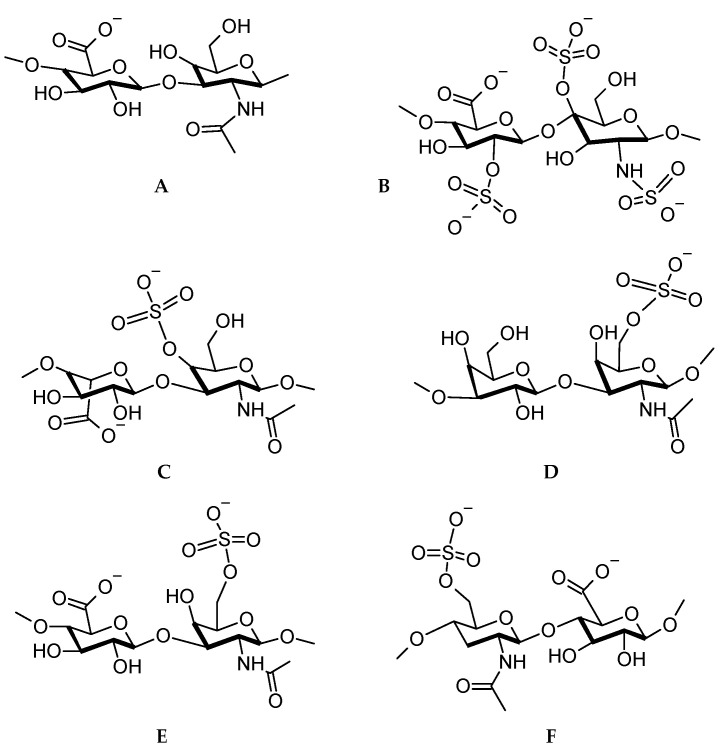
Disaccharide repeat units for (**A**) hyaluronic acid, (**B**) heparan sulfate, (**C**) dermatan sulfate, (**D**) keratan sulfate, (**E**) chondroitin sulfate, (**F**) heparin.

The reader should be able to realize that the LbL technique presents numerous possibilities for the application of polysaccharides as antiviral surfaces. Firstly, the polysaccharides, especially GAGs are abundantly available. Secondly, they can recognize and interact with proteins via a range of intermolecular forces including electrostatic, hydrogen bonding, and hydrophobic bonding [112]. Thirdly, the polysaccharides are biological molecules and in the case of LbL applications, need minimal or no modification to perform their intended function. Fourthly, fairly low quantities of material need to be deposited to coat the substrates. Lastly, they are biocompatible, biodegradable, and most renewable sources of material. It is very apt to illustrate the chemical structures of the GAGs and some other selected polysaccharides at this point. Figure 4 shows the structures based on the official IUPAC recommendations [113].

From Figure 4, it is observed that several anionic functional groups are available for electrostatic interaction, however, numerous hydroxyl and carbonyl groups are also available for hydrogen bonding. The successful application of polysaccharides as antimicrobials now leads us to the possible preventative measures against viruses.

## 6. Targets for LbL Coating and Potential Applications against Coronaviruses as Well as General Antiviral Applications and Challenges

The applications that are discussed in this section are in a sense a crude method of applying highly sophisticated materials and techniques that were developed for in vivo or diagnostic purposes. A so-called nanotrap set of applications has been developed that can be used for the detection of pathogens [114]. These traps rely on the recognitions between antigens and antibodies that were already discussed above. An example of carbohydrate trapping was illustrated for chitosan fibers that were functionalized with sialyllactose, facilitating the capture of influenza viruses via the virus hemagglutinin glycoprotein on its surface [115].

Carbohydrate microarrays have been reviewed comprehensively [116,117] and these were developed to recognize certain pathogens based on glycan-binding properties of lectins. Either glycan or lectin is indicative of a particular pathogen. Numerous analytical methods, for example, fluorescence spectrometry, mass spectrometry, and surface plasmon resonance spectrometry methods are employed for qualitative and quantitative techniques.

### 6.1. Water Sterilization

All humans need to drink water and have access to water to ensure personal hygiene. In the context of the recommendation to wash hands frequently to remove coronavirus and other pathogens, humans need clean water. However, water can easily be contaminated with viruses.

It was proven that representative coronaviruses, mouse hepatitis virus, and transmissible gastrointestinal virus survived for weeks, even in pasteurized, settled sewage. This suggested that sewage and contaminated water sources posed a risk for coronavirus transmission [118]. Currently, numerous research groups have started to establish if SARS-CoV-2 is present in sewage water and traces of the virus have been found in numerous sewage sources [119]. SARS-CoV-2 was also recently detected in the stool of an asymptomatic child [120] and remains in the gastrointestinal tract of pediatric patients for a prolonged period even after clearance from the lungs [121]. It is suggested that the fecal-oral route of transmission should not be excluded as a route of virus transmission [122], however, it is still being investigated if SARS-CoV-2 will spread through contaminated water [123].

An indirect form of LbL coating was effected by the manufacturing of lignin core particles. These lignin cores were coated with prepared cationic lignin particles. Negatively charged cowpea chlorotic mottle viruses and the cationic lignin particles were highly effective in removing the viruses from the water. It is suggested to develop this material into water filtration devices [124].

A microfiltration membrane was developed to filter water and reduce viral counts of a model bacteriophage. The LbL coating of the filters was performed with the synthetic polycation, poly(ethylene imine) which was covalently immobilized onto the poly(ether sulfone) water membrane material. Silver and copper nanoparticles were also coated in the LbL layers, and although an effective antiviral performance was seen, leached markedly into the filtered water [125].

In a proof-of-concept study, silicon wafers were LbL-coated with synthetic polyelectrolytes in bilayers. Antibacterial effects were apparent and also antiviral activity against the H1N1 influenza virus. Although the coated material proved to be 100% bactericidal against waterborne bacteria, it demonstrated 60% virucidal activity against an H1N1 droplet after coating three layers of the polyelectrolyte. However, as the coating was increased to 7.5 bilayers, 100% virucidal activity was observed [126].

LbL coating of nanofiltration membranes with gallic acid and PEI could effectively adsorb selected antibiotics from water. Although not directly related, an antiviral effect can be envisioned by this kind of LbL coating [127].

The successful disinfection of aqueous poliovirus and rotavirus solution was achieved by coating glass slides with synthetic poly(ethylene imine), PEI, cations [128]. Numerous other examples of LbL coatings on surfaces were also shown to be antimicrobial against many waterborne organisms, yet antiviral effects were not reviewed or were simply mentioned briefly. It is, however, apparent that the same intermolecular forces that lead to interaction between surfaces and viruses occur between surfaces and other microorganisms [129,130,131,132].

### 6.2. Air Filtration

As with water, coronaviruses survive on inanimate surfaces [133] that include metal, glass, wood, paper, silicon, surgical gloves, plastic, and non-stick PTFE surfaces for a period of up to nine days on plastic and two days on steel [134]. It was also observed that MERS-CoV could survive in the air for much longer durations than influenza virus strains [135].

However, living tissue such as mucosal membranes harbors these viruses [136]. As with the need for water, humans need to breathe and are, therefore, naturally exposed to all these surfaces. The need for broad-spectrum protection against airborne microbes has been expressed. Three protective measures against airborne spread of viruses have been suggested to ensure the antiviral efficiency of filter materials and lastly, that these materials are not limited to only one type of device or surface [137].

Wearing facial masks is a commonly recommended prophylactic measure against the spread of airborne pathogens [138,139] such as the influenza A virus [140]. It is even recommended that patients suspected of being infected with SARS-CoV-2 should be fitted with masks during surgery or procedure requiring anesthesia [141] and also during transport to the hospital [142]. FFP2 and FFP3/N95 masks offer significant protection against airborne particles and even then the FFP3 masks filter at least 99% of airborne particles, leaving a gap for some particle penetration. These masks also still pose the risk of improper sealing [143].

It has been shown that airborne viruses, for example, norovirus, adenovirus, and torque teno virus can be detected in air samples [144] on for instance PTFE filters that were used to detect SARS-CoV [145], and MERS by the employment of a specialized air sampling instrument [146].

Several viruses, especially respiratory viruses are effectively distributed by airborne droplets [147,148], and very obviously the human coronaviruses such as SARS-CoV [145,149,150], MERS [146,151,152], and SARS-CoV-2 [153,154,155,156] even though some controversy exists regarding respiratory transmission as the only means of distribution [157]. Some examples of viral air filtration are discussed next.

HEPA filters were coated with charged carbon nanotubes. During the filtration study, the nanotubes were shown to be 92% effective in the filtration of a viral bacteriophage, MS2, as a model of viral particle [158]. There are virtually no reports on coated antiviral air filters, let alone employing an LbL coating method to create these masks. These are rapid inactivation action, broad-spectrum inactivation not only against specific strains, and lastly universal application on various media, not just facial masks [159].

The coating of the filter fabric of these masks with PEI achieved more than 99.999% antiviral activity against the T4D bacteriophage virus. These coatings were achieved by dip coating, however, LbL nanocoating was not reported [159].

A disposable polyimide layer has been coated onto porous silicon-based masks, including an N95 mask. The layer proved to be hydrophobic and prevented the accumulation of droplets on the mask almost completely. The film can be peeled off after use and replaced to reuse the mask [160].

A comprehensive review of cellulose-based air filters has shown that cellulose is an effective material to produce antiviral masks. Cellulose ester materials that are anionic are still seen as the most effective antiviral material in filters and again affirms the potential interaction of positively charged spike proteins and negative GAGs. However, LbL nanocoating of cellulose has both been described as a method to produce antiviral filters [161]. In an unrelated application, our research has shown that cellulose can be coated easily employing LbL coating with, for example, chitosan as coating materials [162]. It is proposed that it would be possible to modify a facial mask that is based on a cellulose fabric with antiviral GAGs.

There are indications that LbL nanocoating of air filtration devices can make a significant contribution to the prevention of virus distribution and be very relevant to the prevention of the respiratory variants of human coronaviruses.

### 6.3. Textiles

In addition to wearing facial masks, protective clothing is another important measure against the spread of pathogens. Cotton is one of the most commonly employed textiles used to manufacture clothing.

Cotton fabric, which was ready for use by the textile industry, was LbL nanocoated. The alternating layers comprised of PSS and poly(allylamine) bilayers followed by bilayers of PSS and chitosan. The coated fabric was washed in water to remove the excess polyelectrolyte with or without ultrasonication. The fabric that was washed with ultrasonication produced a more uniformly coated fabric than without. The coated material was exposed to antimicrobial tests and significant antibacterial activity was shown toward *S. aureus* and some activity was still maintained after washing the fabric with a non-ionic detergent. Up to 80% of bactericidal activity was maintained for fabric samples that were washed with ultrasonication compared to samples washed without. The latter samples retained approximately 30% bactericidal effect. Antiviral effects were not investigated [163].

Chitosan-alginate LbL was also reported to impart antibacterial effects on cotton fabric, however, antiviral activity was not determined [164]. Chitosan also afforded antibacterial properties when alternately layered with sulfonated lignin or boric acid onto cotton fabric [165].

Lignin, an abundant product of unexplored commercial value, that is produced from wood pulp during paper manufacturing also impart antibacterial properties to coated textiles [165,166].

Another popular application of the LbL coating of cotton fabrics was to create flame-retardant cloth [167]. Examples include the coating of the material with flame-retardant laponite nanoclay particles [168], cationic starch, and montmorillonite nanoclay particles in alternate layers [169].

Nylon-66, a popular synthetic textile, was also rendered flame-retardant by LbL-nanocoating with cationic chitosan and anionic alginate [170] or anionic phytic acid [171]. Another synthetic textile, polyester, were coated with synthetic PSS and PDDA to impart significant antibacterial properties to the fabric [172,173].

Dual-purpose, environmentally friendly, antibacterial, and flame-retardant layers of poly(hexamethylene guanidine phosphate) and the seaweed-derived alginate could also be coated on cotton fabric [174].

Another example of synthetic polyelectrolyte LbL nanocoating that showed significant antibacterial properties comprises of *N*-halamine-derived copolymer polyelectrolytes, however, the coated cotton fiber had to be immersed in household bleach to elicit antiviral activity. Despite this, the amount of chlorine that was absorbed could be controlled depending on the bilayer thickness [175].

Another approach to producing a coated textile is to coat fibers that are used to weave the textile. Some drug delivery approaches were employed to create chitosan-alginate fibers loaded with a protein drug [176]. Wood pulp fibers were also successfully LbL-coated with chitosan and carboxymethylcellulose [177] and this fiber coating could, for example, lead to paper strengthening [178]. Antibacterial vascular grafts were produced by LbL nanocoating of chitosan and heparin onto the graft materials [179]. It is suggested that pre-coated fibers can be produced and subsequently weaved into an antiviral textile. Again, a scarcity of literature was found that focused on antiviral activity. It provides an “inside-out” method of textile coating instead of coating the finished textile after it was weaved from uncoated fibers. Polysaccharides may again prevail as an effective pre-treatment option prior to weaving fabrics.

Antiviral activities of polysaccharides, especially of unmodified GAGs or uncomplicated naturally derived polysaccharides such as chitosan and pullulan have been reviewed [180], yet at the time of writing, almost no literature could be found where these polysaccharides were LbL-coated onto textiles specifically for an antiviral effect. Antibacterial and improvement of the physical properties of coated textiles seem to be the focus of comprehensive reviews with brief mentioning of antiviral effects [173,181,182].

Despite the lack of explicit LbL nanocoating of textiles, it is illustrated that coating of fabrics could be an effective antiviral countermeasure. Surgical gowns were, for example, laminated with a 3-fold layer of poly(propylene) as the outer layer, PTFE as the middle layer, and a non-woven polyester layer as the innermost layer. PTFE proved essential in curbing viral adhesion and penetration through the fabric [183,184].

A survey of the patent literature also revealed almost no applications of LbL-nanocoating in antiviral settings regarding textiles.

### 6.4. Medical Devices

Seriously ill patients with respiratory distress, and also other patients with other conditions such as cardiovascular disease often require intimate contact with medical devices. Examples of devices include prosthetic heart valves, orthopedic implants, intravascular catheters, artificial hearts, cardiac pacemakers, etc. [185]. Wettability of the surfaces has been shown to play a crucial role in the adhesion of viruses to whichever surface is present. Glass slides were successfully coated with silanes of various hydrophobicity and showed that more hydrophobic surfaces were most efficient in capturing influenza A viruses. This points again to a form of nanotrapping and inactivation of the virus by surface interactions [186]. Glass slides were also LbL coated with polyanionic and polycationic chitosan to eliminate *S. aureus*. It was implied that this LbL process will benefit medical devices with antibacterial properties [187].

Vascular prostheses were coated successfully with alternating layers of human serum albumin and the GAG, heparin, and other carbohydrates such as dextran sulfate. These multilayered contacts prevented the clotting factor, fibrinogen from causing thrombosis which is a serious complication of contact with the medical device [188].

Although not always disclosing full information, self-eliminating coatings on medical devices that comprise mainly of polypeptides were successfully applied to medical devices that could deliver biological substances after implantation into the human body [189], applied to urethral catheters and stents to prevent mucosal tissue of the urethra to block openings of the catheter or stent that will lead to prevention of fluid drainage. These coatings also released antimicrobials although antivirals were not mentioned specifically [190,191]. In a comprehensive review, the future of LbL nanocoating in the medical profession was pointed out, however, antiviral LbL applications were not mentioned [192].

It was interesting to find that LbL-nanocoating of medical devices also revealed very few literature entries and even fewer regarding coating with polysaccharides or antiviral coating.

It was reported that catheter silicon tubes that contained chlorohexidine-containing PEG-micelles were coated with poly(acrylic acid) as polyanion. Again the purpose of this study was to afford antibacterial action to the silicon tubes [193].

Dental implants are another medical device that benefited from antibacterial LbL coating with PSS and PAH. Metronidazole was coated in the final bilayer and this provided the antibacterial effect to prevent periodontal infections [194].

**Figure 5:**
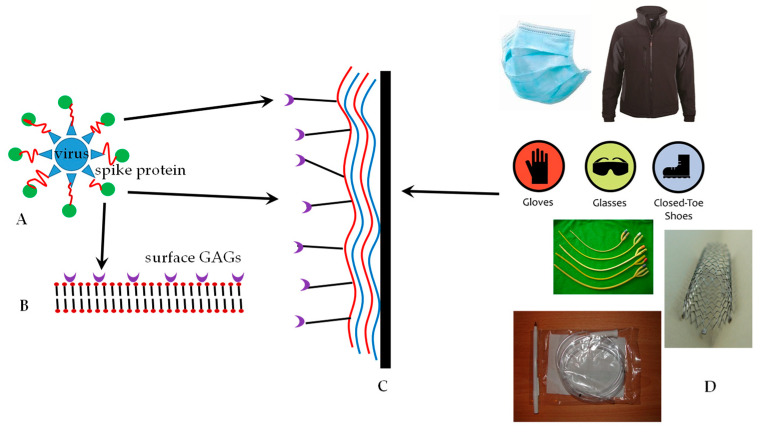
(**A**). The spike proteins of the coronavirus are capable of binding to glycosaminoglycans (GAGs) on the ACE-2 receptor of the lung parenchyma, (**B**,**C**). An ex vivo LbL-nanocoated material acts as a decoy receptor for binding of the spike proteins and inactivating the virus. (**D**). Examples of objects that can be coated are disposable masks, gloves, shoes, catheters, clothing, and intubation tubes. More permanent implants such as vascular stents can also be coated. (All images of (**D**) were sampled under the Creative Commons License Attribution).

It is again apparent, at the time of writing, that antiviral coatings of medical devices have not been the main focus of research or innovation efforts. Figure 5 depicts a summary of our suggested methods of contributing in an almost crude, passive way to curb viral infections.

### 6.5. Challenges to LbL Nanocoating as an Antiviral Measure

It is seen from the literature that LbL nanocoating has been successful against many microorganisms, however, it has not seen significant testing against viruses. It is uncertain how long a surface will remain efficient in trapping viruses, and regular replacement of the materials is probable. Recently [160] it was shown that self-cleaning reusable face masks could be manufactured by coating the textile with hydrophobic silicon dioxide over etched pores of the mask.

On the other hand, it was shown that superhydrophilic chitosan/carboxymethylcellulose LbL coating of silicone ophthalmic devices encouraged the flow of tears and prevented bacterial biofilm formation. The devices also contained antibacterial agents that were released and contributed to the overall effect [195]. Perhaps polysaccharides can be employed in the same way, however, they might require chemical modification. It is also not established if hydro- or hydrophilic coatings will be the most effective.

It has been illustrated that orthopedic implants could be coated with drug-containing niosomes that prevented bacterial adhesion to the implant [196]. Other antibacterial coatings almost always contained antibacterial drugs. Antiviral drugs are not so abundant and other agents might have to be included in the LbL polysaccharide films. These are yet to be identified.

There are no studies that have reported the saturation load that these coatings might accommodate. The disposal of materials that have come in contact with viruses is of concern and protocols for handling this waste should be developed. Not only do they contribute to waste production [197], but also possibly to accumulation of a virus at the place of disposal. Antibacterial textiles have been utilized to manufacture sportswear, underwear, bedding, mattresses, wound dressings, and hospital gowns [198]. These items need laundering in order to reuse and pollute water sources, potentially leading to the accumulation of viruses in the water [199]. Perhaps a disposable product is a better alternative than a reusable product such as illustrated for disposable towels and baths that prevent the accumulation of skin bacteria in patients under nursing care [200].

Clearly many challenges need to be addressed to ensure the effectiveness of these coatings against viruses. The way has, however, been paved by, for example, successful antibacterial and antifungal applications in many settings where humans are exposed to many different surfaces.

## 7. Conclusions and Perspective

Humanity is faced with an unprecedented pandemic. It is the best-documented pandemic that the world has seen and this makes it different from previous, historic pandemics. However, the SARS-CoV-2 pandemic has elicited a concerted, world effort to attempt and curb the effect of the pandemic.

If a cynical view is taken, humanity has to realize that this will not be the last pandemic and that potentially worse pandemics will be seen in the future.

The cusp of antibiotic resistance against many infectious diseases is currently being reached, yet there are even fewer defenses against viral infections. However, nature has provided an abundance of tools, which with human ingenuity and unselfish behavior, can contribute greatly to the prevention of pandemics or at least help to control the spread of these pandemics.

Seen only from an antiviral perspective, many efforts that were launched against other microorganisms can also be effectively applied against viruses. Significant research efforts are focused on the treatment of coronaviruses and these will continue. Of special interest is the employment of antiviral polysaccharide to create virucidal drugs and vaccines. These efforts are aimed primarily at in vivo situations and treatment. It is suggested that many of the in vivo knowledge can be applied to ex vivo, the passive effort against pathogens.

It is known that host cells and viruses interact through glycoprotein and polysaccharide-based interactions. Therefore, the pharmacological effort is attempting to disrupt these interactions or the cellular effects that are seen after the virus and host membrane has indeed merged and the viral mechanism is put into motion.

The human body is a harsh environment and drug delivery and drug development meet these challenges head-on. These include, for example, resistance to absorption of therapeutic agents into the body or degradation of therapeutic agents once they are absorbed into the body.

A clever strategy that is being followed against virus-host cell interaction is to exploit the polysaccharide-lectin recognition system. In vivo efforts have shown that administered polysaccharide-based drugs can serve effectively as decoy binding targets for viruses. Thus, the interaction with membrane-seated viral recognition mechanisms can be circumvented.

Another approach is to induce immunity by presenting polysaccharides or oligosaccharides that represent viral glycoproteins of a specific, or numerous, pathogen(s) to B- and T-cells. These cells will recognize the xenobiotic polysaccharide and activate the immune system cascade and recognize further viruses and eliminate them. If successful, memory cells will be formed and become active when the antigen-antibody, polysaccharide-lectin, interaction occurs in a future infection.

Our suggestion is almost unsophisticated. It is inferred that several surfaces and substrates can be exploited as nanotraps for viruses, outside of the body. It might not be farfetched to suggest that the naturally occurring GAGs will be sufficient to gain positive antiviral results. GAGs are naturally occurring and abundantly available and are suitable to LbL nanocoating in their crude, unrefined state.

It might seem obvious that a layer-by-layer nanocoating strategy will work. However, literature and patent literature surveys have not revealed a significant effort toward antiviral nanocoatings. From the abundant bactericidal reports, it can be deduced that the LbL technique will produce antiviral surfaces. However, we foresee success because surface recognition mechanisms between organisms, hosts, and guests, rely on similar principles and that is protein-polysaccharide interactions.

It is known that numerous polysaccharides have shown antiviral properties and hold significant promise as therapeutic agents. It is suggested to LbL-nanocoat the polysaccharides onto several environmental structures with which humans come into contact daily. We are also optimistic enough to state that researchers in an industry can be successful in this effort since the technique of LbL nanocoating is straightforward, robust, and based on many types of intermolecular forces that can almost guarantee adhesion of materials to a surface of any kind. Numerous examples of LbL nanocoating have been found and described that coat commonly encountered surfaces and produce antibacterial and antifungal actions. Investigation of the antiviral effects of polysaccharide LbL coatings should be investigated and developed. This is an aspect of LbL nanocoating that has not been investigated to a large extent and is a very lucrative option for antiviral research and industrial cooperation. The human, airborne coronavirus are ideal targets for this endeavor. Polysaccharides, in vivo or ex vivo, should be explored for their antiviral applications, especially against coronavirus infections that may be recurring or more frequent in our existence.

## Figures and Tables

**Table 1:** Polysaccharides that have been studied in LbL nanocoating applications.

Polysaccharide	Applications
**GAGs**	
Hyaluronic acid	Formation of pH-sensitive LbL layers for drug delivery applications [91]
	Titanium medical implants coated by LbL with chitosan as a counter polycation for antibacterial effect [92]
	LbL assembled carriers were assembled with chitosan derivatives to control the release of tocopherol and calciferol [93]
Chondroitin sulfate	LbL coating in combination with chitosan was performed to stabilize the controlled release of hydrophobic drugs which agglomerate [94]
	DNA nanoparticles were incorporated via LbL [95]
	Sacrificial calcium carbonate was utilized as a core onto which chondroitin was coated. The hollow capsules were coated with polycations and bovine serum albumin were loaded and released in a pH-dependent way [96]
Heparin	The outer layer of LbL coated particles to improve polysulfone blood compatibility by the anticoagulant effect of heparin [97]
	Coating of a stent with collagen and heparin in LbL multilayers with heparin having anticoagulant effect [98]
	Sacrificial calcium carbonate cores were coated with various polyelectrolytes with heparin or chitosan as outer layers to study dye release from the capsules [99]
**Non-GAGs**	
Chitosan	Proof of cationic chitosan electrostatic interaction for self-assembly [100]
	Review of pH- and sugar-sensing on general drug delivery [101]
	LbL nanocapsule for anti-cancer drug delivery [102]
Alginate	LbL coating of calcium carbonate core loaded with curcumin [103]
	LbL assembly with chitosan. Tamoxifen loaded at different positions in bilayers. [104]
	LbL assembly in combination with dextran that prevented protein sorption to lower fouling of surface [105]
Pectin	Self-assembly with a prodrug-polyelectrolyte for cancer drug delivery [106]
	LbL assembly with poly(allylamine) that included a calcium core loaded with tetracycline [107]
	Investigation of interfacial interaction with bovine serum albumin as an example of polysaccharide-protein binding system [108]
Pullulan	Carboxymethylpullulan assembled with poly(ethylamine) to contain the hydrophobic dye, Nile Red, and test release behavior [109].
	Modified pullulan-derived polyanions assembled with polycations. Provided prove that hydrophobic interactions and not only electrostatic interactions determine self-assembly [110]
Cellulose	Cellulose ethers form hydrogen bonds with poly(acrylic acid) to form LbL films [111]
	Cellulose was coated with various polyelectrolytes to alter the total surface charge density. Higher surface charge density killed more *E. coli* bacteria [85].

## References

[1] Banerjee N., Mukhopadhyay S. (2016). Viral glycoproteins: Biological role and application in diagnosis. Virusdisease.

[2] de Haan C.A., Vennema H., Rottier P.J. (2000). Assembly of the coronavirus envelope: Homotypic interactions between the M proteins. J. Virol..

[3] Coleman C.M., Liu Y.V., Mu H., Taylor J.K., Massare M., Flyer D.C., Glenn G.M., Smith G.E., Frieman M.B. (2014). Purified coronavirus spike protein nanoparticles induce coronavirus neutralizing antibodies in mice. Vaccine.

[4] Sturman L.S., Holmes K.V., Lauffer M.A., Maramorosch K. (1983). The Molecular Biology of Coronaviruses. Advances in Virus Research.

[5] Van Breedam W., Pohlmann S., Favoreel H.W., de Groot R.J., Nauwynck H.J. (2014). Bitter-sweet symphony: Glycan-lectin interactions in virus biology. FEMS Microbiol. Rev..

[6] Varki A. (2017). Biological roles of glycans. Glycobiology.

[7] Watanabe Y., Bowden T.A., Wilson I.A., Crispin M. (2019). Exploitation of glycosylation in enveloped virus pathobiology. Biochim. Biophys. Acta Gen. Subj..

[8] Walls A.C., Park Y.-J., Tortorici M.A., Wall A., McGuire A.T., Veesler D. (2020). Structure, Function, and Antigenicity of the SARS-CoV-2 Spike Glycoprotein. Cell.

[9] Xiao X., Chakraborti S., Dimitrov A.S., Gramatikoff K., Dimitrov D.S. (2003). The SARS-CoV S glycoprotein: Expression and functional characterization. Biochem. Biophys. Res. Commun..

[10] Mathewson A.C., Bishop A., Yao Y., Kemp F., Ren J., Chen H., Xu X., Berkhout B., van der Hoek L., Jones I.M. (2008). Interaction of severe acute respiratory syndrome-coronavirus and NL63 coronavirus spike proteins with angiotensin converting enzyme-2. J. Gen. Virol..

[11] Steinman R.M. (2008). Dendritic cells in vivo: A key target for a new vaccine science. Immunity.

[12] Padron-Regalado E. (2020). Vaccines for SARS-CoV-2: Lessons from Other Coronavirus Strains. Infect. Dis..

[13] Wang D., Tang J., Tang J., Wang L.X. (2015). Targeting N-glycan cryptic sugar moieties for broad-spectrum virus neutralization: Progress in identifying conserved molecular targets in viruses of distinct phylogenetic origins. Molecules.

[14] Mahony J.B., Richardson S. (2005). Molecular diagnosis of severe acute respiratory syndrome: The state of the art. J. Mol. Diagn..

[15] Lu R., Zhao X., Li J., Niu P., Yang B., Wu H., Wang W., Song H., Huang B., Zhu N. (2020). Genomic characterisation and epidemiology of 2019 novel coronavirus: Implications for virus origins and receptor binding. Lancet.

[16] Hao W., Ma B., Li Z., Wang X., Gao X., Li Y., Qin B., Shang S., Cui S., Tan Z. (2020). Binding of the SARS-CoV-2 Spike Protein to Glycans. bioRxiv.

[17] Brojakowska A., Narula J., Shimony R., Bander J. (2020). Clinical Implications of SARS-Cov2 Interaction with Renin Angiotensin System. J. Am. Coll. Cardiol..

[18] Lang J., Yang N., Deng J., Liu K., Yang P., Zhang G., Jiang C. (2011). Inhibition of SARS Pseudovirus Cell Entry by Lactoferrin Binding to Heparan Sulfate Proteoglycans. PLoS ONE.

[19] Chiodo F., Bruijns S.C.M., Rodriguez E., Li R.J.E., Molinaro A., Silipo A., Di Lorenzo F., Garcia-Rivera D., Valdes-Balbin Y., Verez-Bencomo V. (2020). Novel ACE2-Independent Carbohydrate-Binding of SARS-CoV-2 Spike Protein to Host Lectins and Lung Microbiota. bioRxiv.

[20] Hocke A.C., Becher A., Knepper J., Peter A., Holland G., Tönnies M., Bauer T.T., Schneider P., Neudecker J., Muth D. (2013). Emerging Human Middle East Respiratory Syndrome Coronavirus Causes Widespread Infection and Alveolar Damage in Human Lungs. Am. J. Respir. Crit. Care Med..

[21] Nicholls J.M., Poon L.L.M., Lee K.C., Ng W.F., Lai S.T., Leung C.Y., Chu C.M., Hui P.K., Mak K.L., Lim W. (2003). Lung pathology of fatal severe acute respiratory syndrome. Lancet.

[22] van den Brand J.M., Smits S.L., Haagmans B.L. (2015). Pathogenesis of Middle East respiratory syndrome coronavirus. J. Pathol..

[23] Matsuyama S., Ujike M., Morikawa S., Tashiro M., Taguchi F. (2005). Protease-mediated enhancement of severe acute respiratory syndrome coronavirus infection. Proc. Natl. Acad. Sci. USA.

[24] Cheng V.C.C., Lau S.K.P., Woo P.C.Y., Yuen K.Y. (2007). Severe Acute Respiratory Syndrome Coronavirus as an Agent of Emerging and Reemerging Infection. Clin. Microbiol. Rev..

[25] Hamming I., Timens W., Bulthuis M., Lely A., Navis G., van Goor H. (2004). Tissue distribution of ACE2 protein, the functional receptor for SARS coronavirus. A first step in understanding SARS pathogenesis. J. Pathol..

[26] Lin L., Jiang X., Zhang Z., Huang S., Zhang Z., Fang Z., Gu Z., Gao L., Shi H., Mai L. (2020). Gastrointestinal symptoms of 95 cases with SARS-CoV-2 infection. Gut.

[27] Lian N., Xie H., Lin S., Huang J., Zhao J., Lin Q. (2020). Umifenovir treatment is not associated with improved outcomes in patients with coronavirus disease 2019: A retrospective study. Clin. Microbiol. Infect..

[28] Uno Y. (2020). Camostat mesilate therapy for COVID-19. Intern. Emerg. Med..

[29] Heurich A., Hofmann-Winkler H., Gierer S., Liepold T., Jahn O., Pöhlmann S. (2014). TMPRSS2 and ADAM17 cleave ACE2 differentially and only proteolysis by TMPRSS2 augments entry driven by the severe acute respiratory syndrome coronavirus spike protein. J. Virol..

[30] Savarino A., Boelaert J.R., Cassone A., Majori G., Cauda R. (2003). Effects of chloroquine on viral infections: An old drug against today’s diseases. Lancet Infect. Dis..

[31] Devaux C.A., Rolain J.-M., Colson P., Raoult D. (2020). New insights on the antiviral effects of chloroquine against coronavirus: What to expect for COVID-19?. Int. J. Antimicrob. Agents.

[32] Rebeaud M.E., Zores F. (2020). SARS-CoV-2 and the Use of Chloroquine as an Antiviral Treatment. Front. Med..

[33] Bauman J.L., Tisdale J.E. (2020). Chloroquine and Hydroxychloroquine in the Era of SARS–CoV2: Caution on Their Cardiac Toxicity. Pharmacother. J. Hum. Pharmacol. Drug Ther..

[34] Tan E.L.C., Ooi E.E., Lin C.-Y., Tan H.C., Ling A.E., Lim B., Stanton L.W. (2004). Inhibition of SARS coronavirus infection in vitro with clinically approved antiviral drugs. Emerg. Infect. Dis..

[35] Yamamoto N., Yang R., Yoshinaka Y., Amari S., Nakano T., Cinatl J., Rabenau H., Doerr H.W., Hunsmann G., Otaka A. (2004). HIV protease inhibitor nelfinavir inhibits replication of SARS-associated coronavirus. Biochem. Biophys. Res. Commun..

[36] Simmons G., Zmora P., Gierer S., Heurich A., Pöhlmann S. (2013). Proteolytic activation of the SARS-coronavirus spike protein: Cutting enzymes at the cutting edge of antiviral research. Antivir. Res..

[37] Millet J.K., Whittaker G.R. (2015). Host cell proteases: Critical determinants of coronavirus tropism and pathogenesis. Virus Res..

[38] Cinatl J., Michaelis M., Hoever G., Preiser W., Doerr H.W. (2005). Development of antiviral therapy for severe acute respiratory syndrome. Antivir. Res..

[39] Adedeji A.O., Sarafianos S.G. (2014). Antiviral drugs specific for coronaviruses in preclinical development. Curr. Opin. Virol..

[40] Sanders J.M., Monogue M.L., Jodlowski T.Z., Cutrell J.B. (2020). Pharmacologic Treatments for Coronavirus Disease 2019 (COVID-19): A Review. JAMA.

[41] Alexander H.L., Apicella M.A., Christopher E.T. (2005). Carbohydrate Moieties as Vaccine Candidates. Clin. Infect. Dis..

[42] Ahmed S.F., Quadeer A.A., McKay M.R. (2020). Preliminary Identification of Potential Vaccine Targets for the COVID-19 Coronavirus (SARS-CoV-2) Based on SARS-CoV Immunological Studies. Viruses.

[43] Dong S., Sun J., Mao Z., Wang L., Lu Y.-L., Li J. (2020). A guideline for homology modeling of the proteins from newly discovered betacoronavirus, 2019 novel coronavirus (2019-nCoV). J. Med. Virol..

[44] Letko M., Marzi A., Munster V. (2020). Functional assessment of cell entry and receptor usage for SARS-CoV-2 and other lineage B betacoronaviruses. Nat. Microbiol..

[45] Chan J.F.W., Lau S.K.P., To K.K.W., Cheng V.C.C., Woo P.C.Y., Yuen K.-Y. (2015). Middle East Respiratory Syndrome Coronavirus: Another Zoonotic Betacoronavirus Causing SARS-Like Disease. Clin. Microbiol. Rev..

[46] Kirchdoerfer R.N., Cottrell C.A., Wang N., Pallesen J., Yassine H.M., Turner H.L., Corbett K.S., Graham B.S., McLellan J.S., Ward A.B. (2016). Pre-fusion structure of a human coronavirus spike protein. Nature.

[47] Du L., Zhao G., Lin Y., Sui H., Chan C., Ma S., He Y., Jiang S., Wu C., Yuen K.-Y. (2008). Intranasal Vaccination of Recombinant Adeno-Associated Virus Encoding Receptor-Binding Domain of Severe Acute Respiratory Syndrome Coronavirus (SARS-CoV) Spike Protein Induces Strong Mucosal Immune Responses and Provides Long-Term Protection against SARS-CoV Infection. J. Immunol..

[48] Du L., Kou Z., Ma C., Tao X., Wang L., Zhao G., Chen Y., Yu F., Tseng C.T., Zhou Y. (2013). truncated receptor-binding domain of MERS-CoV spike protein potently inhibits MERS-CoV infection and induces strong neutralizing antibody responses: Implication for developing therapeutics and vaccines. PLoS ONE.

[49] Du L., Zhao G., Chan C.C.S., Sun S., Chen M., Liu Z., Guo H., He Y., Zhou Y., Zheng B.-J. (2009). Recombinant receptor-binding domain of SARS-CoV spike protein expressed in mammalian, insect and E. coli cells elicits potent neutralizing antibody and protective immunity. Virology.

[50] Pallesen J., Wang N., Corbett K.S., Wrapp D., Kirchdoerfer R.N., Turner H.L., Cottrell C.A., Becker M.M., Wang L., Shi W. (2017). Immunogenicity and structures of a rationally designed prefusion MERS-CoV spike antigen. Proc. Natl. Acad. Sci. USA.

[51] Kieber-Emmons A., Monzavi-Karbassi B., Kieber-Emmons T. (2020). Antigens: Carbohydrates II. eLS.

[52] Olofsson S., Bergström T. (2005). Glycoconjugate glycans as viral receptors. Ann. Med..

[53] Gulati K., Poluri K.M. (2016). Mechanistic and therapeutic overview of glycosaminoglycans: The unsung heroes of biomolecular signaling. Glycoconj. J..

[54] Kovensky J., Grand E., Uhrig M.L. (2017). Applications of Glycosaminoglycans in the Medical, Veterinary, Pharmaceutical, and Cosmetic Fields. Industrial Applications of Renewable Biomass Products.

[55] Gandhi N.S., Mancera R.L. (2008). The Structure of Glycosaminoglycans and their Interactions with Proteins. Chem. Biol. Drug Des..

[56] Imberty A., Lortat-Jacob H., Pérez S. (2007). Structural view of glycosaminoglycan–protein interactions. Carbohydr. Res..

[57] Ernst B., Magnani J.L. (2009). From carbohydrate leads to glycomimetic drugs. Nat. Rev. Drug Discov..

[58] Martinez J.P., Sasse F., Brönstrup M., Diez J., Meyerhans A. (2015). Antiviral drug discovery: Broad-spectrum drugs from nature. Nat. Prod. Rep..

[59] Lee E., Pavy M., Young N., Freeman C., Lobigs M. (2006). Antiviral effect of the heparan sulfate mimetic, PI-88, against dengue and encephalitic flaviviruses. Antivir. Res..

[60] Lee E.C., Davis-Poynter N., Nguyen C.T.H., Peters A.A., Monteith G.R., Strounina E., Popat A., Ross B.P. (2016). GAG mimetic functionalised solid and mesoporous silica nanoparticles as viral entry inhibitors of herpes simplex type 1 and type 2 viruses. Nanoscale.

[61] Dietrich M.H., Harprecht C., Stehle T. (2017). The bulky and the sweet: How neutralizing antibodies and glycan receptors compete for virus binding. Protein Sci..

[62] Casu B., Naggi A., Torri G. (2002). Chemical Derivatization as a Strategy to Study Structure-Activity Relationships of Glycosaminoglycans. Semin. Thromb. Hemost..

[63] Cagno V., Tseligka E.D., Jones S.T., Tapparel C. (2019). Heparan Sulfate Proteoglycans and Viral Attachment: True Receptors or Adaptation Bias?. Viruses.

[64] Mycroft-West C., Su D., Elli S., Li Y., Guimond S., Miller G., Turnbull J., Yates E., Guerrini M., Fernig D. (2020). The 2019 coronavirus (SARS-CoV-2) surface protein (Spike) S1 Receptor Binding Domain undergoes conformational change upon heparin binding. bioRxiv.

[65] Mycroft-West C.J., Su D., Li Y., Guimond S.E., Rudd T.R., Elli S., Miller G., Nunes Q.M., Procter P., Bisio A. (2020). SARS-CoV-2 Spike S1 Receptor Binding Domain undergoes Conformational Change upon Interaction with Low Molecular Weight Heparins. bioRxiv.

[66] Mycroft-West C.J., Su D., Li Y., Guimond S.E., Rudd T.R., Elli S., Miller G., Nunes Q.M., Procter P., Bisio A. (2020). Glycosaminoglycans induce conformational change in the SARS-CoV-2 Spike S1 Receptor Binding Domain. bioRxiv.

[67] Mycroft-West C.J., Su D., Pagani I., Rudd T.R., Elli S., Guimond S.E., Miller G., Meneghetti M.C.Z., Nader H.B., Li Y. (2020). Heparin inhibits cellular invasion by SARS-CoV-2: Structural dependence of the interaction of the surface protein (spike) S1 receptor binding domain with heparin. bioRxiv.

[68] Dimitrov D.S. (2003). The secret life of ACE2 as a receptor for the SARS virus. Cell.

[69] Milewska A., Zarebski M., Nowak P., Stozek K., Potempa J., Pyrc K. (2014). Human coronavirus NL63 utilizes heparan sulfate proteoglycans for attachment to target cells. J. Virol..

[70] Hofmann H., Pyrc K., van der Hoek L., Geier M., Berkhout B., Pöhlmann S. (2005). Human coronavirus NL63 employs the severe acute respiratory syndrome coronavirus receptor for cellular entry. Proc. Natl. Acad. Sci. USA.

[71] Tamhankar M., Gerhardt D.M., Bennett R.S., Murphy N., Jahrling P.B., Patterson J.L. (2018). Heparan sulfate is an important mediator of Ebola virus infection in polarized epithelial cells. Virol. J..

[72] Chang A., Masante C., Buchholz U.J., Dutch R.E. (2012). Human metapneumovirus (HMPV) binding and infection are mediated by interactions between the HMPV fusion protein and heparan sulfate. J. Virol..

[73] Kesari A.S., Sharkey C.M., Sanders D.A. (2019). Role of heparan sulfate in entry and exit of Ross River virus glycoprotein-pseudotyped retroviral vectors. Virology.

[74] Walls A.C., Tortorici M.A., Frenz B., Snijder J., Li W., Rey F.A., DiMaio F., Bosch B.-J., Veesler D. (2016). Glycan shield and epitope masking of a coronavirus spike protein observed by cryo-electron microscopy. Nat. Struct. Mol. Biol..

[75] Kalia M., Chandra V., Rahman S.A., Sehgal D., Jameel S. (2009). Heparan Sulfate Proteoglycans Are Required for Cellular Binding of the Hepatitis E Virus ORF2 Capsid Protein and for Viral Infection. J. Virol..

[76] Opie S.R., Warrington K.H., Agbandje-McKenna M., Zolotukhin S., Muzyczka N. (2003). Identification of amino acid residues in the capsid proteins of adeno-associated virus type 2 that contribute to heparan sulfate proteoglycan binding. J. Virol..

[77] Hulst M.M., van Gennip H.G., Moormann R.J. (2000). Passage of classical swine fever virus in cultured swine kidney cells selects virus variants that bind to heparan sulfate due to a single amino acid change in envelope protein E(rns). J. Virol..

[78] de Villiers M.M., Otto D.P., Strydom S.J., Lvov Y.M. (2011). Introduction to nanocoatings produced by layer-by-layer (LbL) self-assembly. Adv. Drug Deliv. Rev..

[79] Decher G., Hong J.D., Schmitt J. (1992). Buildup of ultrathin multilayer films by a self-assembly process: III. Consecutively alternating adsorption of anionic and cationic polyelectrolytes on charged surfaces. Thin Solid Film..

[80] Lvov Y., Ariga K., Ichinose I., Kunitake T. (1995). Assembly of Multicomponent Protein Films by Means of Electrostatic Layer-by-Layer Adsorption. J. Am. Chem. Soc..

[81] Sukhorukov G.B., Möhwald H., Decher G., Lvov Y.M. (1996). Assembly of polyelectrolyte multilayer films by consecutively alternating adsorption of polynucleotides and polycations. Thin Solid Film..

[82] Cécius M., Jérôme C. (2011). A fully aqueous sustainable process for strongly adhering antimicrobial coatings on stainless steel. Prog. Org. Coat..

[83] Lin Q.K., Xu X., Wang Y., Wang B., Chen H. (2017). Antiadhesive and antibacterial polysaccharide multilayer as IOL coating for prevention of postoperative infectious endophthalmitis. Int. J. Polym. Mater. Polym. Biomater..

[84] Mokkaphan J., Banlunara W., Palaga T., Sombuntham P., Wanichwecharungruang S. (2014). Silicone surface with drug nanodepots for medical devices. ACS Appl. Mater. Interfaces.

[85] Chen C., Petterson T., Illergård J., Ek M., Wågberg L. (2019). Influence of Cellulose Charge on Bacteria Adhesion and Viability to PVAm/CNF/PVAm-Modified Cellulose Model Surfaces. Biomacromolecules.

[86] Jiang L., Lu Y., Liu X., Tu H., Zhang J., Shi X., Deng H., Du Y. (2015). Layer-by-layer immobilization of quaternized carboxymethyl chitosan/organic rectorite and alginate onto nanofibrous mats and their antibacterial application. Carbohydr. Polym..

[87] Xin S., Li X., Ma Z., Lei Z., Zhao J., Pan S., Zhou X., Deng H. (2013). Cytotoxicity and antibacterial ability of scaffolds immobilized by polysaccharide/layered silicate composites. Carbohydr. Polym..

[88] Pérez-Álvarez L., Ruiz-Rubio L., Azua I., Benito V., Bilbao A., Vilas-Vilela J.L. (2019). Development of multiactive antibacterial multilayers of hyaluronic acid and chitosan onto poly(ethylene terephthalate). Eur. Polym. J..

[89] Wang Y., Hong Q., Chen Y., Lian X., Xiong Y. (2012). Surface properties of polyurethanes modified by bioactive polysaccharide-based polyelectrolyte multilayers. Colloids Surf. B Biointerfaces.

[90] Wu Y., Long Y., Li Q.L., Han S., Ma J., Yang Y.W., Gao H. (2015). Layer-by-Layer (LBL) Self-Assembled Biohybrid Nanomaterials for Efficient Antibacterial Applications. ACS Appl Mater. Interfaces.

[91] Lu B., Luo D., Zhao A., Wang H., Zhao Y., Maitz M.F., Yang P., Huang N. (2019). pH responsive chitosan and hyaluronic acid layer by layer film for drug delivery applications. Prog. Org. Coat..

[92] Valverde A., Perez-Alvarez L., Ruiz-Rubio L., Pacha Olivenza M.A., Garcia Blanco M.B., Diaz-Fuentes M., Vilas-Vilela J.L. (2019). Antibacterial hyaluronic acid/chitosan multilayers onto smooth and micropatterned titanium surfaces. Carbohydr. Polym..

[93] Turcsanyi A., Varga N., Csapo E. (2020). Chitosan-modified hyaluronic acid-based nanosized drug carriers. Int. J. Biol. Macromol..

[94] Strydom S.J., Otto D.P., Stieger N., Aucamp M.E., Liebenberg W., de Villiers M.M. (2014). Self-assembled macromolecular nanocoatings to stabilize and control drug release from nanoparticles. Powder Technol..

[95] Ren K., Ji J., Shen J. (2005). Construction of Polycation-Based Non-Viral DNA Nanoparticles and Polyanion Multilayers via Layer-by-Layer Self-Assembly. Macromol. Rapid Commun..

[96] Zhao Q., Li B. (2008). pH-controlled drug loading and release from biodegradable microcapsules. Nanomedicine.

[97] Mahlicli F.Y., Altinkaya S.A. (2013). Surface modification of polysulfone based hemodialysis membranes with layer by layer self assembly of polyethyleneimine/alginate-heparin: A simple polyelectrolyte blend approach for heparin immobilization. J. Mater. Sci. Mater. Med..

[98] Lin Q., Yan J., Qiu F., Song X., Fu G., Ji J. (2011). Heparin/collagen multilayer as a thromboresistant and endothelial favorable coating for intravascular stent. J. Biomed. Mater. Res. A.

[99] Sun L., Xiong X., Zou Q., Ouyang P., Burkhardt C., Krastev R. (2017). Design of intelligent chitosan/heparin hollow microcapsules for drug delivery. J. Appl. Polym. Sci..

[100] Martins G.V., Mano J.F., Alves N.M. (2010). Nanostructured self-assembled films containing chitosan fabricated at neutral pH. Carbohydr. Polym..

[101] Sato K., Yoshida K., Takahashi S., Anzai J.-I. (2011). pH- and sugar-sensitive layer-by-layer films and microcapsules for drug delivery. Adv. Drug Deliv. Rev..

[102] Wang F., Li J., Tang X., Huang K., Chen L. (2020). Polyelectrolyte three layer nanoparticles of chitosan/dextran sulfate/chitosan for dual drug delivery. Colloids Surf. B Biointerfaces.

[103] Elbaz N.M., Owen A., Rannard S., McDonald T.O. (2020). Controlled synthesis of calcium carbonate nanoparticles and stimuli-responsive multi-layered nanocapsules for oral drug delivery. Int. J. Pharm..

[104] Criado-Gonzalez M., Fernandez-Gutierrez M., San Roman J., Mijangos C., Hernández R. (2019). Local and controlled release of tamoxifen from multi (layer-by-layer) alginate/chitosan complex systems. Carbohydr. Polym..

[105] Wei Y., Hung H.C., Sun F., Bai T., Zhang P., Nowinski A.K., Jiang S. (2016). Achieving low-fouling surfaces with oppositely charged polysaccharides via LBL assembly. Acta Biomater..

[106] Liu Y., Qi Q., Li X., Liu J., Wang L., He J., Lei J. (2017). Self-Assembled Pectin-Conjugated Eight-Arm Polyethylene Glycol–Dihydroartemisinin Nanoparticles for Anticancer Combination Therapy. ACS Sustain. Chem. Eng..

[107] Mihai M., Racovita S., Vasiliu A.L., Doroftei F., Barbu-Mic C., Schwarz S., Steinbach C., Simon F. (2017). Autotemplate Microcapsules of CaCO3/Pectin and Nonstoichiometric Complexes as Sustained Tetracycline Hydrochloride Delivery Carriers. ACS Appl. Mater. Interfaces.

[108] Wang X., Ruengruglikit C., Wang Y.W., Huang Q. (2007). Interfacial interactions of pectin with bovine serum albumin studied by quartz crystal microbalance with dissipation monitoring: Effect of ionic strength. J. Agric. Food Chem..

[109] Guyomard A., Nysten B., Muller G., Glinel K. (2006). Loading and release of small hydrophobic molecules in multilayer films based on amphiphilic polysaccharides. Langmuir.

[110] Guyomard A., Muller G., Glinel K. (2005). Buildup of Multilayers Based on Amphiphilic Polyelectrolytes. Macromolecules.

[111] Zhang X., Lin F., Yuan Q., Zhu L., Wang C., Yang S. (2019). Hydrogen-bonded thin films of cellulose ethers and poly(acrylic acid). Carbohydr. Polym..

[112] Lyklema J., Deschênes L. (2011). The first step in layer-by-layer deposition: Electrostatics and/or non-electrostatics?. Adv. Colloid Interface Sci..

[113] Sharon N. (1986). IUPAC-IUB Joint Commission on Biochemical Nomenclature (JCBN). Nomenclature of glycoproteins, glycopeptides and peptidoglycans. Recommendations 1985. Eur. J. Biochem..

[114] Shafagati N., Patanarut A., Luchini A., Lundberg L., Bailey C., Petricoin E., Liotta L., Narayanan A., Lepene B., Kehn-Hall K. (2014). The use of Nanotrap particles for biodefense and emerging infectious disease diagnostics. Pathog. Dis..

[115] Li X., Wu P., Gao G.F., Cheng S. (2011). Carbohydrate-functionalized chitosan fiber for influenza virus capture. Biomacromolecules.

[116] Park S., Gildersleeve J.C., Blixt O., Shin I. (2013). Carbohydrate microarrays. Chem. Soc. Rev..

[117] Cheng Y., Li M., Wang S., Peng H., Reid S., Ni N., Fang H., Xu W., Wang B. (2010). Carbohydrate biomarkers for future disease detection and treatment. Sci. China Chem..

[118] Casanova L., Rutala W.A., Weber D.J., Sobsey M.D. (2009). Survival of surrogate coronaviruses in water. Water Res..

[119] Mallapaty S. (2020). How sewage could reveal true scale of coronavirus outbreak. Nature.

[120] An T., Zhen-dong T., Hong-ling W., Ya-xin D., Ke-feng L., Jie-nan L., Wen-jie W., Chen Y., Meng-lu Y., Peng L. (2020). Detection of Novel Coronavirus by RT-PCR in Stool Specimen from Asymptomatic Child, China. Emerg. Infect. Dis. J..

[121] Xing Y.-H., Ni W., Wu Q., Li W.-J., Li G.-J., Wang W.-D., Tong J.-N., Song X.-F., Wing-Kin Wong G., Xing Q.-S. (2020). Prolonged viral shedding in feces of pediatric patients with coronavirus disease 2019. J. Microbiol. Immunol. Infect..

[122] Quilliam R.S., Weidmann M., Moresco V., Purshouse H., O’Hara Z., Oliver D.M. (2020). COVID-19: The environmental implications of shedding SARS-CoV-2 in human faeces. Environ. Int..

[123] La Rosa G., Bonadonna L., Lucentini L., Kenmoe S., Suffredini E. (2020). Coronavirus in water environments: Occurrence, persistence and concentration methods - A scoping review. Water Res..

[124] Rivière G.N., Korpi A., Sipponen M.H., Zou T., Kostiainen M.A., Österberg M. (2020). Agglomeration of Viruses by Cationic Lignin Particles for Facilitated Water Purification. ACS Sustain. Chem. Eng..

[125] Sinclair T.R., Patil A., Raza B.G., Reurink D., van den Hengel S.K., Rutjes S.A., de Roda Husman A.M., Roesink H.D.W., de Vos W.M. (2019). Cationically modified membranes using covalent layer-by-layer assembly for antiviral applications in drinking water. J. Membr. Sci..

[126] Wong S.Y., Li Q., Veselinovic J., Kim B.-S., Klibanov A.M., Hammond P.T. (2010). Bactericidal and virucidal ultrathin films assembled layer by layer from polycationic *N*-alkylated polyethylenimines and polyanions. Biomaterials.

[127] Cheng X.Q., Wang Z.X., Guo J., Ma J., Shao L. (2018). Designing Multifunctional Coatings for Cost-Effectively Sustainable Water Remediation. ACS Sustain. Chem. Eng..

[128] Larson A.M., Hsu B.B., Rautaray D., Haldar J., Chen J., Klibanov A.M. (2011). Hydrophobic polycationic coatings disinfect poliovirus and rotavirus solutions. Biotechnol. Bioeng..

[129] Siedenbiedel F., Tiller J.C. (2012). Antimicrobial Polymers in Solution and on Surfaces: Overview and Functional Principles. Polymers.

[130] Hammond P.T. (2011). Engineering materials layer-by-layer: Challenges and opportunities in multilayer assembly. Aiche J..

[131] Ogunsona E.O., Muthuraj R., Ojogbo E., Valerio O., Mekonnen T.H. (2020). Engineered nanomaterials for antimicrobial applications: A review. Appl. Mater. Today.

[132] Bassyouni M., Abdel-Aziz M.H., Zoromba M.S., Abdel-Hamid S.M.S., Drioli E. (2019). A review of polymeric nanocomposite membranes for water purification. J. Ind. Eng. Chem..

[133] Chin A.W.H., Chu J.T.S., Perera M.R.A., Hui K.P.Y., Yen H.-L., Chan M.C.W., Peiris M., Poon L.L.M. (2020). Stability of SARS-CoV-2 in different environmental conditions. Lancet Microbe.

[134] Kampf G., Todt D., Pfaender S., Steinmann E. (2020). Persistence of coronaviruses on inanimate surfaces and their inactivation with biocidal agents. J. Hosp. Infect..

[135] Pyankov O.V., Bodnev S.A., Pyankova O.G., Agranovski I.E. (2018). Survival of aerosolized coronavirus in the ambient air. J. Aerosol. Sci..

[136] Kampf G. (2020). Potential role of inanimate surfaces for the spread of coronaviruses and their inactivation with disinfectant agents. Infect. Prev. Pract..

[137] Rubino I., Choi H.-J. (2017). Respiratory Protection against Pandemic and Epidemic Diseases. Trends Biotechnol..

[138] Yang P., Seale H., Raina MacIntyre C., Zhang H., Zhang Z., Zhang Y., Wang X., Li X., Pang X., Wang Q. (2011). Mask-wearing and respiratory infection in healthcare workers in Beijing, China. Braz. J. Infect. Dis..

[139] Cheng V.C.-C., Wong S.-C., Chuang V.W.-M., So S.Y.-C., Chen J.H.-K., Sridhar S., To K.K.-W., Chan J.F.-W., Hung I.F.-N., Ho P.-L. (2020). The role of community-wide wearing of face mask for control of coronavirus disease 2019 (COVID-19) epidemic due to SARS-CoV-2. J. Infect..

[140] Weiss M.M., Weiss P.D., Weiss D.E., Weiss J.B. (2007). Disrupting the Transmission of Influenza A: Face Masks and Ultraviolet Light as Control Measures. Am. J. Public Health.

[141] Wax R.S., Christian M.D. (2020). Practical recommendations for critical care and anesthesiology teams caring for novel coronavirus (2019-nCoV) patients. Can. J. Anesth. /J. Can. D’anesthésie.

[142] Peng P.W.H., Ho P.-L., Hota S.S. (2020). Outbreak of a new coronavirus: What anaesthetists should know. Br. J. Anaesth.

[143] Lam S.C., Lee J.K.L., Yau S.Y., Charm C.Y.C. (2011). Sensitivity and specificity of the user-seal-check in determining the fit of N95 respirators. J. Hosp. Infect..

[144] Carducci A., Verani M., Lombardi R., Casini B., Privitera G. (2011). Environmental survey to assess viral contamination of air and surfaces in hospital settings. J. Hosp. Infect..

[145] Booth T.F., Kournikakis B., Bastien N., Ho J., Kobasa D., Stadnyk L., Li Y., Spence M., Paton S., Henry B. (2005). Detection of Airborne Severe Acute Respiratory Syndrome (SARS) Coronavirus and Environmental Contamination in SARS Outbreak Units. J. Infect. Dis..

[146] Kim S.-H., Chang S.Y., Sung M., Park J.H., Bin Kim H., Lee H., Choi J.-P., Choi W.S., Min J.-Y. (2016). Extensive Viable Middle East Respiratory Syndrome (MERS) Coronavirus Contamination in Air and Surrounding Environment in MERS Isolation Wards. Clin. Infect. Dis..

[147] Knight V. (1980). Viruses as Agents Of Airborne Contagion. Ann. N. Y. Acad. Sci..

[148] Yu I.T., Li Y., Wong T.W., Tam W., Chan A.T., Lee J.H., Leung D.Y., Ho T. (2004). Evidence of airborne transmission of the severe acute respiratory syndrome virus. N. Engl. J. Med..

[149] Tsai Y.-H., Wan G.-H., Wu Y.-K., Tsao K.-C. (2006). Airborne Severe Acute Respiratory Syndrome Coronavirus Concentrations in a Negative-Pressure Isolation Room. Infect. Control. Hosp. Epidemiol..

[150] Tong T.R. (2005). Airborne Severe Acute Respiratory Syndrome Coronavirus and Its Implications. J. Infect. Dis..

[151] Al-Tawfiq J.A., Memish Z.A. (2014). Middle East respiratory syndrome coronavirus: Transmission and phylogenetic evolution. Trends Microbiol..

[152] Rasmussen S.A., Gerber S.I., Swerdlow D.L. (2015). Middle East Respiratory Syndrome Coronavirus: Update for Clinicians. Clin. Infect. Dis..

[153] Wilson N.M., Norton A., Young F.P., Collins D.W. (2020). Airborne transmission of severe acute respiratory syndrome coronavirus-2 to healthcare workers: A narrative review. Anaesthesia.

[154] Guan L., Zhou L., Zhang J., Peng W., Chen R. (2020). More awareness is needed for severe acute respiratory syndrome coronavirus 2019 transmission through exhaled air during non-invasive respiratory support: Experience from China. Eur. Respir. J..

[155] Wilson M.E., Chen L.H. (2020). Travellers give wings to novel coronavirus (2019-nCoV). J. Travel Med..

[156] Cook T.M. (2020). Personal protective equipment during the coronavirus disease (COVID) 2019 pandemic—A narrative review. Anaesthesia.

[157] Lewis D. (2020). Is the coronavirus airborne? Experts can’t agree. Nature.

[158] Park K.T., Hwang J. (2014). Filtration and inactivation of aerosolized bacteriophage MS2 by a CNT air filter fabricated using electro-aerodynamic deposition. Carbon.

[159] Tiliket G., Sage D.L., Moules V., Rosa-Calatrava M., Lina B., Valleton J.M., Nguyen Q.T., Lebrun L. (2011). A new material for airborne virus filtration. Chem. Eng. J..

[160] El-Atab N., Qaiser N., Badghaish H., Shaikh S.F., Hussain M.M. (2020). Flexible Nanoporous Template for the Design and Development of Reusable Anti-COVID-19 Hydrophobic Face Masks. ACS Nano.

[161] Junter G.A., Lebrun L. (2017). Cellulose-based virus-retentive filters: A review. Rev. Environ. Sci. Biotechnol..

[162] Strydom S.J., Otto D.P., Liebenberg W., Lvov Y.M., de Villiers M.M. (2011). Preparation and characterization of directly compactible layer-by-layer nanocoated cellulose. Int. J. Pharm..

[163] Joshi M., Khanna R., Shekhar R., Jha K. (2011). Chitosan nanocoating on cotton textile substrate using layer-by-layer self-assembly technique. J. Appl. Polym. Sci..

[164] Gomes A., Mano J., Queiroz J., Gouveia I. (2012). Layer-by-Layer Deposition of Antibacterial Polyelectrolytes on Cotton Fibres. J. Polym. Environ..

[165] Safi K., Kant K., Bramhecha I., Mathur P., Sheikh J. (2020). Multifunctional modification of cotton using layer-by-layer finishing with chitosan, sodium lignin sulphonate and boric acid. Int. J. Biol. Macromol..

[166] Juikar S.J., Vigneshwaran N. (2017). Microbial production of coconut fiber nanolignin for application onto cotton and linen fabrics to impart multifunctional properties. Surf. Interfaces.

[167] Chang S., Slopek R.P., Condon B., Grunlan J.C. (2014). Surface Coating for Flame-Retardant Behavior of Cotton Fabric Using a Continuous Layer-by-Layer Process. Ind. Eng. Chem. Res..

[168] Li Y.-C., Schulz J., Grunlan J.C. (2009). Polyelectrolyte/Nanosilicate Thin-Film Assemblies: Influence of pH on Growth, Mechanical Behavior, and Flammability. ACS Appl. Mater. Interfaces.

[169] Choi K., Seo S., Kwon H., Kim D., Park Y.T. (2018). Fire protection behavior of layer-by-layer assembled starch-clay multilayers on cotton fabric. J. Mater. Sci..

[170] Kumar Kundu C., Wang W., Zhou S., Wang X., Sheng H., Pan Y., Song L., Hu Y. (2017). A green approach to constructing multilayered nanocoating for flame retardant treatment of polyamide 66 fabric from chitosan and sodium alginate. Carbohydr. Polym..

[171] Laufer G., Kirkland C., Morgan A.B., Grunlan J.C. (2012). Intumescent multilayer nanocoating, made with renewable polyelectrolytes, for flame-retardant cotton. Biomacromolecules.

[172] Smith R.J., Moule M.G., Sule P., Smith T., Cirillo J.D., Grunlan J.C. (2017). Polyelectrolyte Multilayer Nanocoating Dramatically Reduces Bacterial Adhesion to Polyester Fabric. ACS Biomater. Sci. Eng..

[173] Murugesh Babu K., Ravindra K.B. (2015). Bioactive antimicrobial agents for finishing of textiles for health care products. J. Text. Inst..

[174] Fang F., Chen X., Zhang X., Cheng C., Xiao D., Meng Y., Ding X., Zhang H., Tian X. (2016). Environmentally friendly assembly multilayer coating for flame retardant and antimicrobial cotton fabric. Prog. Org. Coat..

[175] Cerkez I., Kocer H.B., Worley S.D., Broughton R.M., Huang T.S. (2011). N-Halamine Biocidal Coatings via a Layer-by-Layer Assembly Technique. Langmuir.

[176] Liao I.C., Wan A.C.A., Yim E.K.F., Leong K.W. (2005). Controlled release from fibers of polyelectrolyte complexes. J. Control. Release.

[177] Kramer R.K., Guimarães F.E.G., Carvalho A.J.F. (2019). Wood pulp fiber modification by layer-by-layer (LBL) self-assembly of chitosan/carboxymethyl cellulose complex: Confocal microscopy characterization. J. Mol. Liq..

[178] Rudi H., Saedi H., Kermanian H. (2019). Fabrication of self-assembled polysaccharide multilayers on broke chemi-mechanical pulp fibers: Effective approach for paper strength enhancement. Polym. Test..

[179] Li L., Wang X., Li D., Qin J., Zhang M., Wang K., Zhao J., Zhang L. (2020). LBL deposition of chitosan/heparin bilayers for improving biological ability and reducing infection of nanofibers. Int. J. Biol. Macromol..

[180] Smelcerovic A., Knezevic-Jugovic Z., Petronijevic Z. (2008). Microbial Polysaccharides and their Derivatives as Current and Prospective Pharmaceuticals. Curr. Pharm. Des..

[181] Yetisen A.K., Qu H., Manbachi A., Butt H., Dokmeci M.R., Hinestroza J.P., Skorobogatiy M., Khademhosseini A., Yun S.H. (2016). Nanotechnology in Textiles. ACS Nano.

[182] Sehulster L.M. (2015). Healthcare Laundry and Textiles in the United States: Review and Commentary on Contemporary Infection Prevention Issues. Infect. Control. Hosp. Epidemiol..

[183] Parthasarathi V., Thilagavathi G. (2015). Development of plasma enhanced antiviral surgical gown for healthcare workers. Fash. Text..

[184] Parthasarathi V., Thilagavathi G. (2013). Developing antiviral surgical gown using nonwoven fabrics for health care sector. Afr. Health Sci..

[185] Rodrigues L.R. (2011). Novel Approaches to avoid Microbial Adhesion onto Biomaterials. J. Biotechnol. Biomater..

[186] Mannelli I., Reigada R., Suárez I., Janner D., Carrilero A., Mazumder P., Sagués F., Pruneri V., Lakadamyali M. (2016). Functionalized Surfaces with Tailored Wettability Determine Influenza A Infectivity. ACS Appl. Mater. Interfaces.

[187] Bulwan M., Wójcik K., Zapotoczny S., Nowakowska M. (2012). Chitosan-Based Ultrathin Films as Antifouling, Anticoagulant and Antibacterial Protective Coatings. J. Biomater. Sci. Polym. Ed..

[188] Brynda E., Houska M., Jiroušková M., Dyr J.E. (2000). Albumin and heparin multilayer coatings for blood-contacting medical devices. J. Biomed. Mater. Res..

[189] Esfand R., Santerre J.P., Ernsting Mark J., Wang Vivian Z., Tjahyadi S. (2008). Self-Eliminating Coatings. European Patent.

[190] Erbey J.R., Tucker B.J., Upperco J.L. (2019). Coated and/or Impregnated Ureteral Catheter or Stent and Method. U.S. Patent.

[191] Fulmer P.A., Wynne J.H. (2011). Development of Broad-Spectrum Antimicrobial Latex Paint Surfaces Employing Active Amphiphilic Compounds. ACS Appl. Mater. Interfaces.

[192] Zhao S., Caruso F., Dähne L., Decher G., De Geest B.G., Fan J., Feliu N., Gogotsi Y., Hammond P.T., Hersam M.C. (2019). The Future of Layer-by-Layer Assembly: A Tribute to ACS Nano Associate Editor Helmuth Möhwald. ACS Nano.

[193] Tambunlertchai S., Srisang S., Nasongkla N. (2017). Development of antimicrobial coating by layer-by-layer dip coating of chlorhexidine-loaded micelles. J. Mater. Sci. Mater. Med..

[194] Gentile P., Frongia M.E., Cardellach M., Miller C.A., Stafford G.P., Leggett G.J., Hatton P.V. (2015). Functionalised nanoscale coatings using layer-by-layer assembly for imparting antibacterial properties to polylactide-co-glycolide surfaces. Acta Biomater..

[195] Park S., Park J., Heo J., Lee S.-E., Shin J.-W., Chang M., Hong J. (2018). Polysaccharide-based superhydrophilic coatings with antibacterial and anti-inflammatory agent-delivering capabilities for ophthalmic applications. J. Ind. Eng. Chem..

[196] Dwivedi A., Mazumder A., Nasongkla N. (2018). Layer-by-layer nanocoating of antibacterial niosome on orthopedic implant. Int. J. Pharm..

[197] Yasin S., Sun D. (2019). Propelling textile waste to ascend the ladder of sustainability: EOL study on probing environmental parity in technical textiles. J. Clean. Prod..

[198] Windler L., Height M., Nowack B. (2013). Comparative evaluation of antimicrobials for textile applications. Environ. Int..

[199] Sun G., McCarthy B.J. (2011). 8-Disposable and reusable medical textiles. Textiles for Hygiene and Infection Control.

[200] Matsumoto C., Nanke K., Furumura S., Arimatsu M., Fukuyama M., Maeda H. (2019). Effects of disposable bath and towel bath on the transition of resident skin bacteria, water content of the stratum corneum, and relaxation. Am. J. Infect. Control..

